# Deciphering the peptidome of urine from ovarian cancer patients and healthy controls

**DOI:** 10.1186/1559-0275-11-23

**Published:** 2014-06-02

**Authors:** Christopher R Smith, Ihor Batruch, Josep Miquel Bauça, Hari Kosanam, Julia Ridley, Marcus Q Bernardini, Felix Leung, Eleftherios P Diamandis, Vathany Kulasingam

**Affiliations:** 1 Department of Clinical Biochemistry, University Health Network, Toronto, ON, Canada; 2 Samuel Lunenfeld Research Institute, Department of Pathology and Laboratory Medicine, Mount Sinai Hospital, Toronto, ON, Canada; 3 Servei d’Anàlisis Clíniques, Hospital Universitari Son Espases, Palma de Mallorca, Spain; 4 Department of Psychosocial Oncology and Palliative Care, Princess Margaret Cancer Center, University Health Network, Toronto, ON, Canada; 5 Division of Palliative Care, Department of Community and Palliative Medicine, University of Toronto, Toronto, ON, Canada; 6 Department of Obstetrics and Gynecology, University of Toronto, Toronto, ON, Canada; 7 Department of Laboratory Medicine and Pathobiology, University of Toronto, 200 Elizabeth Street, Room 3 EB 362A, Toronto, ON M5G 2C4, Canada

**Keywords:** Biomarker, Early diagnosis, Mass spectrometry, Ovarian cancer, Urine peptidome

## Abstract

**Background:**

Ovarian cancer (OvCa) is the most lethal gynecological malignancy. The emergence of high-throughput technologies, such as mass spectrometry, has allowed for a paradigm shift in the way we search for novel biomarkers. Urine-based peptidomic profiling is a novel approach that may result in the discovery of noninvasive biomarkers for diagnosing patients with OvCa. In this study, the peptidome of urine from 6 ovarian cancer patients and 6 healthy controls was deciphered.

**Results:**

Urine samples underwent ultrafiltration and the filtrate was subjected to solid phase extraction, followed by fractionation using strong cation exchange chromatography. These fractions were analyzed using an Orbitrap mass spectrometer. Over 4600 unique endogenous urine peptides arising from 713 proteins were catalogued, representing the largest urine peptidome reported to date. Each specimen was processed in triplicate and reproducibility at the protein (69-76%) and peptide (58-63%) levels were noted. More importantly, over 3100 unique peptides were detected solely in OvCa specimens. One such promising biomarker was leucine-rich alpha-2-glycoprotein (LRG1), where multiple peptides were found in all urines from OvCa patients, but only one peptide was found in one healthy control urine sample.

**Conclusions:**

Mining the urine peptidome may yield highly promising novel OvCa biomarkers.

## Background

Ovarian cancer (OvCa) is the fifth largest killer and the most lethal gynecological malignancy, accounting for approximately 3% of all new cancer cases in 2012 [1]. The reason for the high fatality rate is that the disease is often diagnosed in advanced stages (stage III and IV) when the cancer has metastasized to other organs. The 5-year survival rate for patients with advanced disease is 10-30%, whereas those diagnosed in earlier stages have a 5-year survival rate that exceeds 90% [2]. Carbohydrate antigen 125 (CA125) is the most widely used and clinically accepted serum marker for OvCa. Despite its widespread use for monitoring therapeutic response in OvCa patients, CA125 is a poor marker for early diagnosis due to frequent false positive and false negative results. Other markers have shown utility in the detection of OvCa such as HE4, osteopontin, CA 15–3 and CA19-9; however, none of these proteins have shown efficacy for early detection of OvCa [3]. For these reasons, efforts to discover novel OvCa biomarkers continue to date.

The investigation of the peptidome, or the low-molecular weight proteome, of biological fluids relevant to OvCa is an emerging field. It is hypothesized that metabolic activity increases in tandem with the progression of malignancy and consequently, protease activity increases as well. Thus, endogenous peptides are generated, some of which may be secreted into the surrounding environment where they can theoretically be detected and used to monitor disease. Furthermore, the progression of malignancy is also associated with the degradation of adhesion and cell-to-cell junction proteins and this may also be another source of endogenous peptides with diagnostic potential. It is well known that peptides play complex regulatory roles in many biological processes, such as intercellular signaling [4-9]. Current proteomic approaches to biomarker discovery, utilizing the power of mass spectrometry (MS), make it possible to delineate the endogenous peptidomes of various bodily fluids.

One such biofluid that may be suited for OvCa diagnosis is urine. The analysis of urine plays a central role in clinical diagnostics due to its availability in almost all patients and its simplicity of collection non-invasively. Urine can be collected in large volumes and its excretion is a normal and necessary function. This is in contrast to other bodily fluids such as blood, where collection is more invasive, prone to collection artifacts and where the activation of proteases (especially the coagulation cascade) generates many proteolytic breakdown products [10]. Urine is known to be relatively stable, likely due to the fact that it is ‘stored’ for hours in the bladder and therefore, proteolytic degradation may be complete at the time of voiding [11]. Urine has also been shown to contain a diverse set of proteins/peptides, where approximately 70% of all urinary proteins originate from the kidney, and 30% are derived from the plasma, making it an attractive fluid for biomarker discovery [11].

Although peptidomics is still in its infancy, there have already been a few studies that have reported on the utility of peptides for OvCa diagnostics. Fredolini et al. reported 59 peptide markers that were unique to OvCa patients compared to patients with benign gynecologic conditions [12]. On the contrary, Timms *et al*. recently reported that MALDI MS peptide profiling was unable to accurately diagnose OvCa from healthy controls, though the endogenous peptides could provide some diagnostic insight [13]. Needless to say, greater characterization of the endogenous peptidome of various biospecimens related to OvCa is needed to truly assess whether or not peptide-based biomarkers are clinically useful. Since the first serum peptidomic study of OvCa [14], multiple studies in several body fluids have pointed out a wide array of low-molecular-weight proteins and peptides with high disease-specific information for different types of cancer [15,16].

In this study, we were able to identify several thousands of endogenous peptides in the urine of OvCa patients and healthy controls. Our work represents the largest urine peptidomic study to date for any disease type. In addition, there are no studies in the literature looking at urine peptidomics and OvCa. This makes our peptidomics study of major importance in the pursuit of early diagnostic biomarkers for this malignancy. A number of our identified peptides may have value as candidate OvCa biomarkers and could be targeted for further validation in the future.

## Results and discussion

### Optimization of sample preparation for urine peptidome identification

To perform an in-depth peptidomic analysis on urine, it is essential to have a procedure that is robust and comprehensive. To accomplish this, each step of the procedure was examined carefully and alternative strategies were applied before selecting the “optimal” procedure that yielded the greatest number of peptides reproducibly. Method development and evaluation were performed using the second morning urine of a single donor, spiked with known concentrations of synthetic heavy peptides from five proteins [IL8 (3 peptides), VEGF (3 peptides), IGFBP2 (4 peptides), HER2 (4 peptides) and VIP (1 peptide)] at known concentrations (ranging from 2–1000 pmol). Methods were considered to be superior if more peptides were identified (both endogenous and spiked).

One of the first steps considered when performing peptidomic analysis on urine was the low protein content of urine, in relation to other fluids such as serum. As a result, it was essential to examine methods for urine concentration. Centrifugal filter units are inexpensive, easy to use and can rapidly separate the protein and peptide components. In this study, the Millipore Amicon Ultracel concentrators (5 and 10 kDa MWCO) and Sartorius Stedium Vivaspin 20 mL concentrators (5 and 10 kDa MWCO) were evaluated and a greater number of peptides were identified with the Vivaspin 20 mL 10 kDa MWCO unit (data not shown). Using this centrifugal filter, 10–30 mL of filtrate was required prior to further treatment and injection on the mass spectrometer. Next, the most appropriate solid-phase extraction column was selected by examining two different cartridges: C8 (Varian SPEC) and Oasis HLB. The Oasis cartridges were able to identify more peptides than the Varian C8 SPE cartridges (data not shown). However, in the eluted fraction of both cartridges, a ‘brown substance’ that co-purified with the endogenous peptide fraction was present. This substance was partially removed by further processing the sample with ethyl acetate, as reported previously [17]. Partial removal of this unknown substance led to an approximate two fold increase in peptide identification (data not shown).

**Figure 1:**
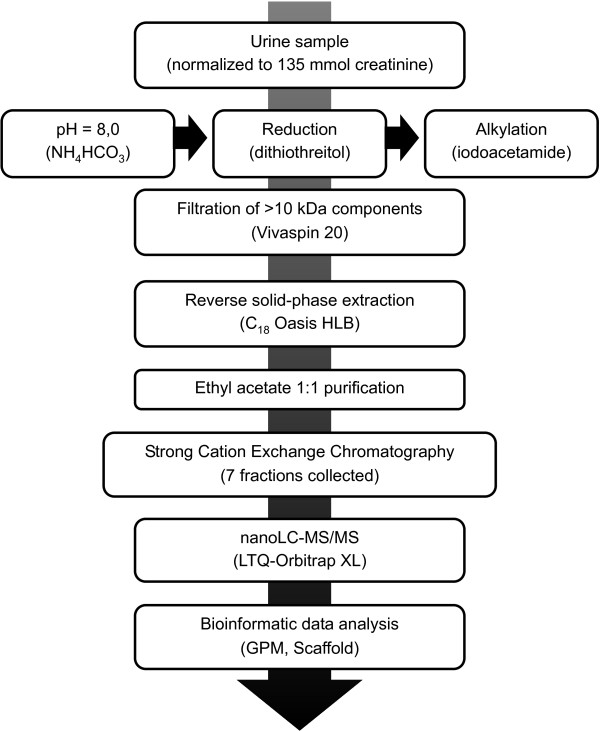
**Outline of experimental workflow (peptidomic analysis).** The workflow consisted of an optimized sample processing procedure, followed by strong cation exchange and reverse-phase chromatography coupled online to an LTQ-Orbitrap mass spectrometer, and subsequent data analysis.

When urine samples were not reduced or alkylated, very few peptides with cysteine (cys) residues were detected (data not shown). This was unexpected since the frequency of cys in the IPI 3.71 human database is 3%, and the frequency of cysteine in peptides that were detected in various published urine *proteomes* was also 3% [18,19]. By examining urine *peptidomes* from the literature, it was noted that they had an unusually low level of cysteines [20-23]. This finding led us to examine this anomaly in greater detail. After analyzing the cys content of various other peptidomes, it became readily apparent that those studies that reduced and alkylated their fluids prior to peptide extraction [24,25] had higher cys content than those that did not [20-23]. This finding was of no surprise to us and the exclusion of cysteine-containing peptides from the urine peptidome by not reducing or alkylating may be detrimental. Many biologically active peptides, such as insulin and endothelin, contain disulfide bridges which not only determine the functional structure of the proteins but also often protect them from proteolysis. Additionally, some of the antimicrobial endogenous peptides such as defensins, are bonded into specific structures via disulfide bridges [24]. In this study, we were able to identify several cysteine-containing peptides that have known biological importance such as the granulins, uteroglobin and hepcidin (Additional file 1: S1A and S1B). The benefit of reduction and alkylation of samples in the identification of endogenous peptides has also been reported elsewhere [26]. Figure 1 depicts the final sample preparation procedure that was followed for deciphering the urine peptidome, based on the optimization steps outlined above.

### Optimization of bioinformatics for urine peptidome identification

Once the sample preparation was optimized, the ability of the GPM search engine to detect endogenous peptides was examined. The same concatenated forward-random database, as well as the same .mgf files generated from extract_msn was used. The resultant .xml files were analyzed in Scaffold to calculate and set the false-discovery rate (FDR) at 1%. The effect of the inclusion or exclusion of oxidized proline (oxP) in terms of the total number of peptides and proteins identified was also examined. These results showed that GPM did not show a large difference in the number of proteins and peptides identified with the inclusion or exclusions of oxP. For example, without oxidized proline, 739 proteins and 4539 peptides were identified. With oxidized proline, 713 proteins and 4607 peptides were identified. Based on the above results, the final bioinformatic searches were performed with GPM and the following modifications: oxP and oxidized methionine (oxM).

### Deciphering the ovarian cancer urine peptidome

**Figure 2:**
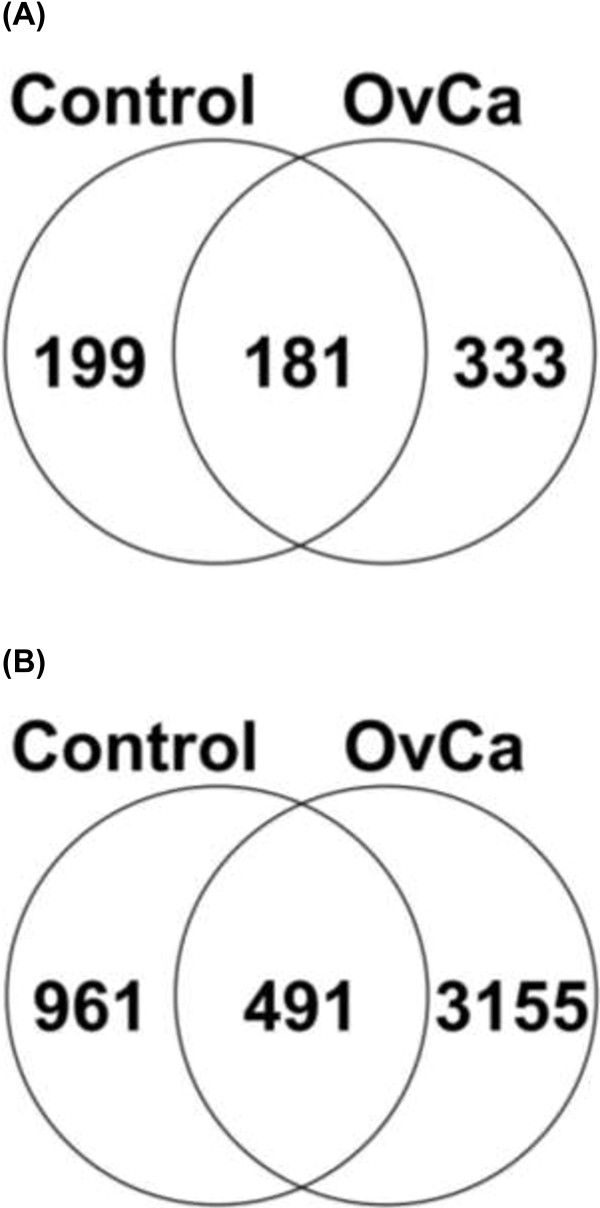
**Overlap of the total proteins (A) and peptides (B) identified in control (n = 6) and ovarian cancer (OvCa) (n = 6) samples.** Each sample was processed in triplicate. Over 300 unique proteins were found in the OvCa urine and over 3100 peptides were unique to OvCa compared to healthy controls.

In conventional bottom-up proteomic experiments, proteins are digested with trypsin, and each protein is identified by fully tryptic peptides. Tryptic peptides have C-terminal basic amino acids that are protonated under acidic conditions. These protonated amino acids assist in peptide fragmentation and subsequent bioinformatic identification. Since the protease(s) responsible for the production of endogenous urine peptides are unknown, the data required the use of no enzyme specificity ([X]|[X]), and very few variable modifications (to limit database size). Sparse use of variable modifications is disadvantageous to endogenous peptide identification as many of these peptides are known to be modified [26]. Despite these limitations, from the 6 OvCa and 6 control urine specimens examined in this study, we were able to identify 4607 unique peptides, including known endogenous urine peptides, originating from 713 protein precursors (Figure 2, Additional file 1: S1 and S2). The Scaffold file used in this study can be found in Supplementary Information S2 and a free Scaffold 2.06 viewer can be downloaded from http://www.peptideatlas.org/PASS/PASS00204 (follow link ftp://PASS00204:ZH3882n@ftp.peptideatlas.org/). Figure 2 (A) and (B) displays the overlap between the proteins and unique peptides identified in the control and OvCa samples, respectively. The control samples consisted of 380 proteins and 1452 unique peptides, while the OvCa cohort consisted of 514 proteins and 3646 unique peptides. A large fraction of the peptides were uniquely identified in the OvCa samples and this could have possibly resulted from reported higher protease activity in malignant cells [4-9]. The list of 333 unique proteins to OvCa is provided in Additional file 2: S3A. The list of 3155 unique peptides identified solely from OvCa specimensare provided in Additional file 2: S3B.

The results of the individual sample analysis yielded between 268 to 1933 unique peptides and 84–374 proteins per specimen (Table 1). To our knowledge, this repository represents the largest collection of endogenous urinary peptides to date. Most importantly, it presents the only OvCa urine peptidome in the literature [27].

**Table 1:** Number (triplicate analysis) of proteins and peptides found in urine of 6 ovarian cancer patients (OvCa) and 6 healthy controls

**Sample**	**Proteins**	**Standard deviation**	**Peptides**	**Standard deviation**
OvCa_1	192	8	1005	34
OvCa_2	84	18	313	51
OvCa_3	103	2	367	4
OvCa_4	374	15	1933	53
OvCa_5	118	21	482	52
OvCa_6	116	9	659	6
Control_1	125	17	382	81
Control_2	121	3	377	10
Control_3	91	13	268	38
Control_4	171	7	526	122
Control_5	223	4	756	22
Control_6	109	3	357	39
All	713		4607	

Number of proteins and peptides are the sum of triplicate analysis with a false-discovery rate (FDR) of 1%. The average number of proteins identified in the OvCa and control samples are 165 and 140, whereas the average number of unique peptides are 793 and 444, respectively. “All” represents the total number of non-redundant proteins and peptides identified in all 12 specimens analyzed.

**Figure 3:**
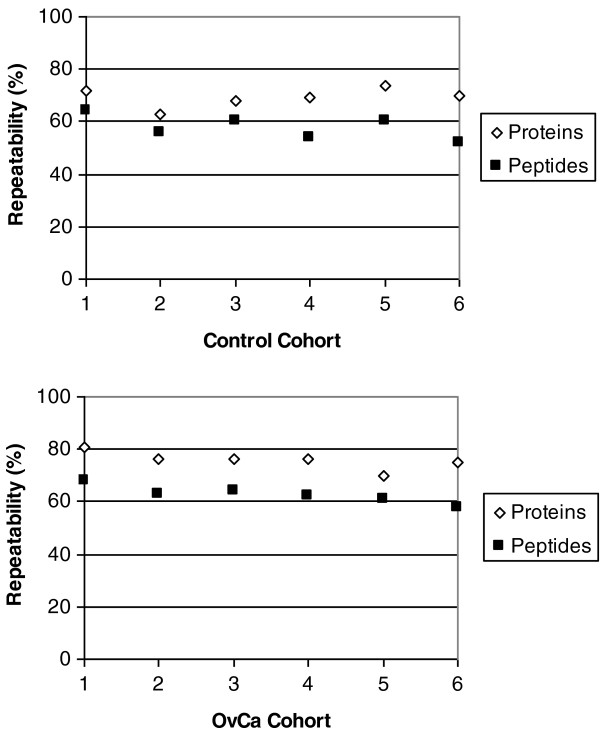
**Protein and peptide repeatability.** To assess repeatability, we examined the overlapping fraction of identified protein or peptide in pairs of biological replicates. The percentage of repeatability for proteins (open diamond) vs peptides (closed box) for each sample is shown.

It should be noted that all urine samples were processed and analyzed in triplicate. The repeatability of our methodology is shown in Figure 3, which highlights the overlap at the protein and peptide level for all OvCa and healthy control urine samples that were independently processed in triplicate. On average, 69-76% reproducibility was observed at the protein level and 58-63% was observed at the peptide level for both cases and controls. These values were based on protein and/or peptides list from pairs of technical replicates. Our results are consistent with the literature showing higher repeatability and reproducibility for proteins than for peptides [28]. This highlights the importance of processing each sample in triplicate or more to truly decipher as much of the peptidome as possible. Our results indicate that there is a large inter-individual variability in peptide/protein content between OvCa and control urine samples (Table 1). Since we performed triplicate analysis of each sample (Figure 3), we can be confident that this variability is due to physiological/biological differences in urine content rather than the technical aspects of sample preparation. In a recent study of variability of the normal urinary proteome by Nagaraj et al., it was shown that intrapersonal and interpersonal variability contributed 46% and 47% to total variability, respectively [29]. Overall variability is also greater in OvCa patients than controls and this could be a result of differences in medical conditions of the OvCa patients. Identification of more peptides and higher inter-individual variability in peptide identifications in OvCa urine suits the hypothesis which states that malignant cells have higher metabolic and protease activity than normal cells [4-9]

### Comparison of ovarian urine proteome and peptidome to other publications

Fredolini et al. compared the proteome of 20 serum samples from patients with early-stage epithelial OvCa with 20 samples from patients diagnosed with benign gynecological conditions [12]. A total of 59 proteins were found to be over-expressed in OvCa compared to the control cohort. Out of these 59 proteins, our study identified 16 of these proteins (which represents 27% overlap). Additional file 3: S4 contains the list of overlapping proteins for each publication examined here. It should be noted that the objective of our study was not to decipher the proteome of urine OvCa and thus, it is not surprising that more proteins were not overlapping with Fredolini et al. Another study, by Lopez et al., looked at the serum proteome of 110 healthy individuals and 453 patients with OvCa (including stages I-IV) [30]. The authors identified 160 proteins that were overexpressed in ovarian cancer compared to healthy controls. Among the 160 proteins, our study identified 15 of these candidates, yielding an overlap of 9.4%. Three proteins (Fibrinogen beta chain, Keratin, type I cytoskeletal 9 and Transgelin-2) were identified by all 3 studies. A recent publication has implicated transgelin-2 to have a role in the development and progression of brain metastases from gynecological malignancies [31]. Lastly, Siwy et al. published a paper examining the human urinary peptide database [27]. This consisted of 114 proteins and 787 peptides. It should be mentioned that OvCa was not included in the list of diseases examined. Nonetheless, our study identified 63 of these proteins and 283 peptides.

### Lack of collagen peptides

The most notable difference between peptides identified in this study and others is the lack of identified collagen peptides. On a percentage basis, collagen peptides made up 9% of the total identified peptides in our study as compared to 87% [32], and 74% [20] in other studies, respectively. These finding led Good et al. to state that “Collagen fragments, especially fragments of collagen alpha-1 (I) chain, appear to be the major constituents of urinary peptides” [20]. We were unable to determine the root cause for this large discrepancy. One possible reason for the similarity in collagen frequency in the previously mentioned studies could be that similar methods were used(same research group). We noted also that these studies used several variable modifications, including oxP, in the bioinformatic processing of their data. It has been shown that proline residues are oxidized in collagen [33], and a review of these studies demonstrated the identification of peptides with oxP. When we processed our data with Mascot (another search engine) and included oxP and oxM (to make the bioinformatic processing of our data more similar to other studies) we found collagen peptide content to be 7%. However, despite mimicking the bioinformatics to the previous studies, we were unable to identify such a high percentage of collagen peptides in our present study.

### Leucine-rich alpha-2-glycoprotein: a potential ovarian cancer biomarker

**Figure 4:**
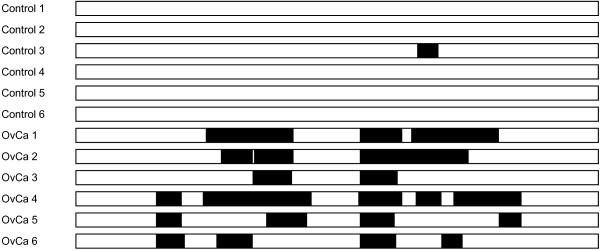
**Peptides identified in the LRG1 protein.** A schematic representation of the LRG1 protein and the highlighted areas reveal the peptides identified per sample.

One of the highly promising biomarkers that emerged from this study was Leucine-rich alpha-2-glycoprotein (LRG1). Interestingly, with the exception of one peptide being identified in Control sample #3 (GKDLLLPQPDLRY), LRG1 was detected only in OvCa samples (present in all 6 OvCa urine specimens). Figure 4 displays a schematic representation of the LRG1 protein and the highlighted areas reveal the peptides that were identified per sample. It is evident that no single identical peptide was consistently identified in all samples (OvCa and control). A large number of these peptides in OvCa samples appear to be generated by exopeptidase activity of a ‘common peptide core’ creating a ladder like effect (data not shown). Similar findings have been seen in other peptidomes deciphered to date [34,35]. The LRG1 protein has previously been suggested to be an OvCa biomarker. First, it was identified in OvCa ascites fluid by our group [36]. In addition, it was found to be upregulated in the sera of OvCa patients by Chen et al. using 2-dimensional gel electrophoresis followed by MALDI-MS identification [37]. It was also identified by another group based on a serum-peptidomics approach using nanoparticles [12]. These discovery results showing LRG1 as a potential novel biomarker for OvCa were verified using an immunoassay (ELISA) on serum and tissues of affected women [38]. In the latter study, a statistically significant two-fold increase in serum LRG1 was found compared to healthy controls. Such differential results were also obtained when comparing mRNA levels in ovarian tissue samples. However, one limitation of this approach was that the area under the curve (AUC) for LRG1 and the AUC of the combined markers (LRG1 + CA125) were not statistically different from the AUC of CA125 alone. Recently, LRG1 was found to be enriched in the urine of patients with appendicitis [39], as well as the serum of individuals with lung cancer [40], and heart failure [41]. It has also been suggested as a biomarker of ulcerative colitis [42]. These results demonstrate elevated expression of LRG1 protein in several disorders, and therefore LRG1 *protein* levels may not be suitable for use as a specific diagnostic marker of OvCa. On the other hand, unique LRG1 endogenous peptides found in the urine of ovarian cancer patients may hold diagnostic value. The function of LRG1 is unknown; however, it has been suggested that it may play a role in cell adhesion [43,44].

Further examination of peptides identified from the LRG1 protein showed that all OvCa samples contain peptides from the same core region with the presence of Arginine (Arg) at positions 191 and 209 in the sequences. Therefore, treatment of the OvCa samples with trypsin could produce the common proteotyptic peptide ^192^TLDLGENQLETLPPDLLR^209^ which can be used to quantitate the level of LRG1 peptide in other, independent urine sample cohorts. Future studies will focus on quantitation of the tryptic LRG1 peptide TLDLGENQLETLPPDLLR in OvCa urine samples.

## Conclusions

In this study, we developed an optimized procedure to identify endogenous peptides in urine from OvCa patients and healthy individuals. Using this strategy, a large number of endogenous urine peptides (>4600 peptides) were identified, representing the largest repository of urine peptidomics to date. This database contains valuable information that will enable future researchers to identify novel OvCa peptide biomarkers, as over 3000 unique peptides were identified solely in OvCa urines compared to healthy control urine specimens. The high abundance of LRG1 peptides in the OvCa sample cohort, and near absence in controls, in conjunction with the previous identification of LRG1 protein in various diseases, suggest that future validation of LRG1 peptides by using quantitative selected reaction monitoring assays in larger numbers of urines is warranted.

## Methods

### Sample collection

Second morning urine samples from 6 late-stage, serous ovarian cancer patients and 6 age-matched healthy women were obtained after written informed consent and institutional ethics board approval. After collection, samples were aliquoted and immediately frozen at −20°C until further analysis. One aliquot was used to perform urinalysis dipstick testing (Clinitek Atlas), and urine creatinine measurements using clinical laboratory analyzers (Abbott Architect). Histological examination of the tumor tissue samples obtained at surgery classified all six patients as having a serous subtype (stage IV). Statistical analysis of the control group (n = 6) and OvCa patients (n = 6) indicated that the two groups were not significantly different in terms of age.

### Sample preparation

Urine samples were thawed at 37°C and vortexed to re-suspend any precipitate. All specimens (cases and controls) were processed in triplicate and in parallel, using the same lot of reagents and columns, to minimize variability or bias in sample preparation. The samples were centrifuged at 2000 g. Urine volumes were normalized with respect to 135 mmol of creatinine per sample (approximately 10–20 mL per individual). The pH of the urines was adjusted to 8.0 by the addition of solid ammonium bicarbonate. A final concentration of 2 mmol/L (mM) of dithiothreitol (DTT) from Sigma-Aldrich was added and the samples incubated at room temperature for 30 min before the addition of a final concentration of 4 mM iodoacetamide (Sigma-Aldrich). The urine was then concentrated with Vivaspin 20 mL 10 kDa cutoff membranes (Sartorius Stedim Biotech), according to the manufacturer’s instructions (concentrators were flushed with 20 mL water prior to use). The concentrator flow through was acidified by drop wise addition of formic acid to pH 4.0. Following the molecular weight enrichment for endogenous peptides, the samples were passed through a hydrophilic-lipophilic-balanced reversed-phase cartridge (Oasis HLB). The cartridge (1 cc (30 mg); Waters cat# WAT094225) was pre-equilibrated with 1 mL 90% acetonitrile (ACN), 0.1% formic acid and 0.02% TFA. The cartridge was washed with 15 mL buffer A (5% ACN, 0.1% formic acid and 0.02% TFA) and the acidified sample was loaded. The cartridge was washed with 15 mL of buffer A. Peptides were eluted by adding 700 uL of 60% ACN, 0.1% formic acid, 0.02% TFA. The eluted fraction was mixed with an equal volume of ethyl acetate and centrifuged at 17 000 g for 5 minutes. The upper layer was discarded and the sample was reduced to a volume of approximately 200 uL via speed vac.

### Strong cation exchange chromatography

An equal volume of mobile phase A (0.26 M formic acid in 5% ACN) was added to the sample and injected into a PolySULFOETHYL A column with a 200-Å pore size and diameter of 5 μm (The Nest Group, Inc) containing a hydrophilic, anionic polymer (poly-2-sulfethyl aspartamide). A 1 hour separation was performed on an HPLC system (Agilent 1100) using a mobile phase B containing 0.26 M formic acid in 5% ACN and 1 M ammonium formate. The eluate was monitored at a wavelength of 280 nm. Seven fractions per sample were collected at a flow rate of 200 μL/min.

### Mass spectrometry

The fractions were desalted by using an Omix C18 pipette tip (Varian) and eluted in 5 μL of buffer B (70% acetonitrile, 0.1% formic acid). After elution, 80 μL of buffer A (0.1% formic acid) was added to each sample and 40 μL were loaded onto a 2-cm C18 trap column, packed with Varian Pursuit (5 μm C18), using the EASY-nLC system (Proxeon Biosystems). Peptides were eluted from the trap column onto a resolving 5-cm analytical C18 column packed with Varian Pursuit (3 μm C18) with an 8 μm tip (New Objective). This LC setup was coupled online to an LTQ-Orbitrap XL (Thermo Fisher Scientific) mass spectrometer using a nanoelectrospray ionization source (Proxeon Biosystems). Each fraction underwent a 54-min gradient, and eluted peptides were subjected to 1 full scan (350–2000 m/z) in the Orbitrap at 60 000 resolution, followed by top 6 data-dependent MS/MS scans in the linear ion trap. With the use of charge-state screening and preview mode, unassigned charge states were rejected.

### Data analysis

Raw files were used to generate Mascot Generic Files (MGF) through extract_msn on Mascot Daemon (version 2.2.2). Once generated, MGFs were searched with X!Tandem (Global Proteome Machine Manager; version 2006.06.01) to confer peptide identifications. Searches were conducted against the non-redundant Human IPI database (v.3.71) which contains a total of 173,490 forward and randomized protein sequences and using the following parameters for GPM: no enzyme ([X]|[X]) cleavages, 50 missed cleavage sites allowed, 7 ppm precursor ion mass tolerance, 0.4 Da fragment ion mass tolerance, fixed modifications of carbamidomethylation of cysteines, and variable modification of oxidation of methionines (oxM) and/or prolines (oxP). The X!Tandem (XML files) were then integrated through the Scaffold 2 software (version 2.06; Proteome Software Inc., Portland, Oregon). False-discovery rates (FDR) were calculated as the number of peptides identified by the randomized reverse database divided by the total number of identified peptides. To achieve a peptide FDR of 1%, X!Tandem –Log(ExpectScores) peptide scores of 2.2 or greater were accepted.

## Abbreviations

OvCa: Ovarian cancer; LRG-1: Leucine-rich alpha-2-glycoprotein; CA125: Carbohydrate antigen 125; GPM: Global proteome machine; oxM: Oxidized methionine; oxP: Oxidized proline; cys: Cysteine; FDR: False discovery rate.

## Competing interests

The authors have declared no conflict of interest.

## Authors’ contributions

CRS, IB, JMB, HK and FL carried out the experimental protocol and data analysis. JR and MQB aided in the sample collection and data analysis. EPD and VK participated in the design of the study and aided in data analysis. All authors participated in the writing of the manuscript and have read/approved the final draft.

## Supplementary Material

Additional file 1 **Peptide and protein report.** Click here for file

Additional file 2 List of proteins and peptides detected solely in OvCa urine. Click here for file

Additional file 3 **List of overlapping proteins with other publications.** Click here for file
